# Toward Multi-Dimensional Depression Assessment: EEG-Based Machine Learning and Neurophysiological Interpretation for Diagnosis, Severity, and Cognitive Decline

**DOI:** 10.3390/brainsci16020139

**Published:** 2026-01-28

**Authors:** Farhad Nassehi, Asuhan Zupan, Aykut Eken, Sinan Yetkin, Osman Erogul

**Affiliations:** 1 Biomedical Engineering Department, TOBB University of Economics and Technology, 06560 Ankara, Turkey; aykuteken@etu.edu.tr (A.E.); erogul@etu.edu.tr (O.E.); 2 Department of Mental Health and Diseases, Atatürk State Hospital, 67030 Zonguldak, Turkey; asuhanpar@hotmail.com; 3 Department of Psychiatry, Health Science University, Gülhane Medical Application and Research Center, 06510 Ankara, Turkey; snnyetkin@gmail.com

**Keywords:** depression, machine learning, feature selection, severity, cognitive impairment

## Abstract

**Background/Objectives:** Depressive disorder (DD) is a prevalent psychiatric condition often diagnosed through subjective self-reports, which can be time-consuming and lead to inaccurate assessments. To enhance diagnostic precision, integrating Electroencephalography (EEG) with machine learning (ML) has gained attention as an objective approach for DD diagnosis and severity assessment. **Methods:** We propose an interpretable EEG-based ML framework that integrates optimized functional connectivity features, including Coherence, Phase Lag Index (PLI), and Granger causality, to explore EEG-based functional connectivity patterns in individuals clinically diagnosed with depressive DD and to model symptom severity and cognitive vulnerability. The identified biomarkers provide a promising foundation for developing objective, clinically actionable decision-support tools in psychiatric care. Feature selection was performed using the Neighborhood Component Analysis (NCA) method, and biomarkers were identified through statistical tests. **Results**: The highest classification performance (97.66% ± 2.05%accuracy, 99.20% ± 1.10% sensitivity, 95.91% ± 4.66% specificity, 98.00% ± 1.02% f1-score, and 0.95 ± 0.48 MCC) was achieved using 21 NCA-selected features with a KNN (K = 9) classifier. The best severity assessment (*r*^2^ = 0.89 ± 0.10, MSE = 3.96 ± 17.05) and cognitive impairment prediction (*r*^2^ = 0.89 ± 0.06, MSE = 0.23 ± 0.45) were obtained using an ANN regressor with 20 and 17 NCA-selected features, respectively. **Conclusions**: Our approach outperforms previous EEG-based ML models in DD classification and severity prediction using fewer features. Notably, this is the first study to use EEG connectivity features to predict patients’ severity and cognitive impairment in DD. Coherence and PLI values from frontal and temporal pathways across the alpha, beta, and gamma sub-bands may serve as critical biomarkers for DD diagnosis, severity assessment, and prediction of cognitive impairment.

## 1. Introduction

Depressive disorder (DD) is a highly prevalent mental health condition that affects individuals across diverse age groups and socio-economic backgrounds. The lifetime prevalence of DD is estimated at approximately 16%, with a one-year prevalence of around 6.7% globally. DD is generally characterized by mood and cognitive dysfunction, along with significant psychosocial impairment, which can persist for weeks to years. An individual is diagnosed as DD when they exhibit a persistent depressed mood, anhedonia, feelings of guilt or worthlessness, or suicidal thoughts [1]. According to the fifth edition of the Diagnostic and Statistical Manual (DSM-5), a diagnosis of DD requires the presence of at least five symptoms that significantly impair social and occupational functioning, with at least one being either a depressed mood or anhedonia. These symptoms must be present for at least 2 weeks. The Beck Depression Inventory (BDI) and the Hamilton Depression Rating Scale (HAMD) are the most commonly used questionnaires to assess DD. In addition to these tests, the Montreal Cognitive Assessment (MOCA) is used to evaluate the cognitive dysfunction in DD patients.

However, the standard protocol for diagnosing DD relies on subjective self-report questionnaires and interviews. With recent developments in neuroimaging, such as electroencephalography (EEG), functional magnetic resonance imaging (fMRI), and functional near-infrared spectroscopy (fNIRS), objective assessment of DD might be possible. Several neuroimaging studies have examined the neurophysiological changes associated with DD [2,3,4]. Among neuroimaging techniques, EEG is widely used to understand neural representations of psychiatric disorders due to its portability, low cost, accessibility, and high temporal resolution. In addition to conventional EEG response patterns such as Event-Related Potentials (ERPs) and Event-Related Oscillations (EROs), Functional connectivity (FC) is used to estimate spatiotemporal relationships in neural dynamics. FC can be defined as the interdependence between brain regions and EEG electrodes, quantified by calculating correlations or coherences between signals from different EEG electrodes, which reflect the connections between brain regions [5]. Using FC analysis for each EEG sub-band may lead to understanding the varying connections between depressed brain regions and highlighted reasons for the disorder and related deficits. EEG studies report that DD patients exhibit altered FC, compared with healthy subjects, although the direction and nature of these changes differ across studies. However, the most common changes are reported in the theta, alpha, and beta sub-bands, and in frontal, temporal, and parietal regions, depending on the network. Phase Lag Index (PLI) and Phase Locking Value (PLV) are the most popular techniques that are used in EEG FC studies [6]. The PLI values in DD patients increased in the theta and beta sub-bands and decreased in the alpha sub-band, especially in frontal and left-hemisphere regions. Interhemispheric PLI values showed significant differences [7]. Studies that surveyed PLV changes in DD patients report declines in right temporal and left occipital, posterior parietal, and right temporal PLV across theta, alpha, and beta sub-bands, and increases in PLV in left temporal–left occipital, frontal–temporal, and parietal–occipital circuits [8]. Other studies report increased PLV in theta, alpha, and beta bands, especially in the prefrontal and left frontal–temporal regions, with the most pronounced changes in the alpha band [9,10]. The combination of neuroimaging data with machine learning (ML) and deep learning (DL) approaches to detect depression and predict its severity has grown significantly among researchers over the last decade. Integrated ML and DL approaches in the diagnostic phase of mental disorders, such as depression, lead to a reduction in time to diagnosis and increased accuracy and determination of the diagnostic phase. The major challenge in integrating EEG features with ML/DL algorithms is the high dimensionality of the feature space, which leads to increased algorithmic complexity. In addition to this problem, the high number of features may include irrelevant or redundant ones, leading to reduced accuracy.

Several studies used EEG and artificial intelligence approaches to either classify DD or predict DD severity and cognitive impairments. Nassibi et al. proposed an algorithm to detect depression. In this study, they used neighborhood component analysis (NCA) to identify optimal EEG electrodes and features and reported maximum performance of 91.8% accuracy, 93.5% specificity, and 90% sensitivity using 6 features [11]. Zhang et al. also combined EEG features from Fp1, Fp2, and Fpz electrodes and speech signal features and reported 76.40% accuracy, 74.04% F1-score, 70.78% sensitivity, and 81.52% specificity [12]. In a multimodal study, Xinfang et al. classified depression using a combination of ERP-EEG, eye-tracking, and galvanic skin response signals, achieving 79.63% accuracy, 76.67% precision, and 85.19% recall [13]. Mumtaz et al. achieved 98% accuracy, 99.9% sensitivity, 95% specificity, and an F1-score of 0.97 in detecting depression using EEG band-power and Alpha band interhemispheric asymmetry features. They achieved this performance by employing 100 electrode pairs [14]. Movahed et al. also used functional connectivity approaches to detect depression. They reached 93.3% ± 8.2% accuracy, 93.9% ± 7.5% sensitivity, and 92.8% ± 9.2% specificity rates using 171 features [15]. Earl et al. also used EEG signals recorded during resting-state and emotional task sessions. They used a functional connectivity approach to analyze EEG signals. They achieved 60% accuracy using only 52 selected features extracted from resting-state signals [16]. Chen et al. also conducted a study to diagnose primary DD using the PLI and phase-locking value (PLV) methods. They reached 88.73% accuracy with 90.67% sensitivity and 86% specificity when 29 selected features from principal component analysis (PCA), K-means, and mutual information (PKM) were used as inputs to the convolutional neural network (CNN) classifier [17]. Table 11 summarizes and compares the literature surveys. In addition to the aforementioned studies, which combine EEG and artificial intelligence (AI) to detect DD, this combination is also used in another study that focused on predicting BDI scores to assess DD severity. Kang et al. conducted a study to predict the severity of DD based on BDI scores using power analysis of EEG signals and a CNN regressor. They reported a Root Mean Square Error (RMSE) of 3.46 for the prediction task [18]. This study does not use a connectivity-based approach to extract features. Hashempour and colleagues also used a CNN trained on raw EEG signals to predict DD severity based on the BDI score. They reported an MSE of 5.64 ± 1.6 and a mean absolute error (MAE) of 1.73 ± 0.27 when using eyes-open (EO) EEG signals [19]. This study also does not use any feature extraction method. The current study aims to leverage differences in neural mechanisms to estimate disease severity using FC analysis methods.

Several studies use FC features and artificial intelligence for automatic classification of DD; however, few use FC to predict DD severity based on the BDI score. Another deficiency in the literature is that MOCA scores for DD patients have not been estimated from EEG signals using machine learning algorithms. Accurate prediction of the BDI score enables continuous, non-invasive monitoring of symptom severity, facilitating timely treatment adjustments. Also, the MOCA score prediction may lead to early detection of cognitive decline, which is common in DD patients and often may be overlooked in standard psychiatric evaluations.

**Figure 1:**
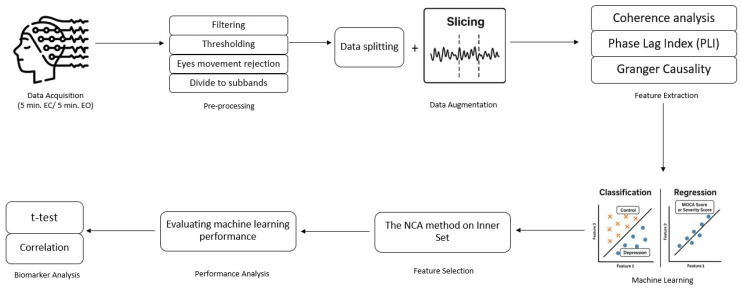
Scheme of study.

In this study, our main objective is to (1) investigate the optimal EEG resting-state recording type (eyes closed (EC)/eyes open (EO)) for identifying DD and predicting its severity, based on classification and regression results. (2) Develop an EEG-based decision-support and screening framework using FC features derived from resting-state EEG recordings of individuals clinically diagnosed with depressive disorder; to evaluate how well these features discriminate between groups using machine learning approaches with at least 90% accuracy rate and 0.90 AUC rate; to identify the most informative pathways and features selected by the NCA method; and to examine their potential biomarker relevance through statistical analysis (*t*-test, *p* < 0.05). (3) Predict the severity of DD patients according to BDI score using selected FC features by the NCA method and investigate the abilities of these pathways and features as biomarkers. (4) Predict the MOCA score that represents the cognitive state of DD patients using selected FC features by the NCA method, propose optimal pathways and features, and investigate the abilities of these pathways and features as biomarkers. In both 3. and 4., our criteria are an R-squared (*r*^2^) greater than 0.80, an MSE less than 10 in the regression step, and a meaningful Pearson correlation coefficient (*p* < 0.05). For this purpose, we aim to develop an engineering-driven EEG analysis framework that advances the state of the art in functional connectivity-based depression assessment. By systematically integrating multiple connectivity metrics (coherence, PLI, Granger causality), optimizing feature selection with NCA, and combining classification and regression models, the proposed approach aims to achieve both high accuracy and clinical interpretability, offering a promising avenue for objective clinical diagnostics (Figure 1). Furthermore, by extending the framework to predict depression severity and cognitive impairment, this work aims to provide an essential step towards real-world applications of EEG-based decision support systems in psychiatry. We aimed not only to enhance diagnostic accuracy but also to explore potential digital biomarkers that reflect clinically relevant dimensions of depression, including affective severity and cognitive dysfunction. To the best of our knowledge, it is the first study that tries to propose a framework to classify DD patients, predict the severity of the disorder and cognitive impairment score of patients, and introduce the optimal pathways and features as biomarkers.

## 2. Materials and Methods

### 2.1. Participants

Participants were recruited from the registries of the Health Sciences University, the Gülhane Faculty of Medicine, and the Gülhane Training and Research Hospital, Department of Mental Health and Diseases. All participants were at least 18 years old and provided written informed consent before participation. Inclusion criteria comprised an age range of 18–45 years and right-handedness, assessed using the Edinburgh Handedness Inventory. Participants were excluded if they had any comorbid psychiatric diagnosis according to DSM-5 or if they had used psychotropic medication prescribed for psychiatric conditions other than depressive disorder within the last month. Based on these criteria, 22 patients (16 females, 6 males) diagnosed with depressive disorder (DD) according to DSM-5 criteria were included. All patients were experiencing their first depressive episode and had not yet initiated antidepressant treatment at the time of EEG data acquisition. In addition, 25 healthy control subjects (17 females, 8 males) with no psychiatric history, matched for age and gender, were enrolled. All participants underwent 5 min resting-state EEG recordings under eyes-closed (EC) and eyes-open (EO) conditions and completed the Beck Depression Inventory (BDI) and Montreal Cognitive Assessment (MOCA). The experimental protocol was approved by the Health Sciences University Scientific Research Ethics Committee (Decision File Number: 2022-366).

Table 1 presents the demographic and test score information for two groups, expressed as mean ± standard deviation.

### 2.2. Data Acquisition

5 min EO and EC resting state EEG signals were recorded at the Mental Health and Diseases Department of Gülhane Training and Research Hospital. All signals were recorded using the UPM-PLUS (GRASS TECHNOLOGIES, Rockland, MA, USA) Polysomnography (PSG) device. The resting state EEG data were recorded using 16 Ag/AgCl EEG electrodes (C3, T3, C4, T4, Fp1, Fp2, F3, F4, F7, F8, P3, P4, T5, T6, O1, O2) on the scalp based on the 10–20 system with 200 Hz sampling rate with A1 and A2 reference electrodes placed on earlobes. To detect eye movements, vertical electrooculography (EOG) signals from both eyes were recorded during all EEG sessions. EEG and EOG signals were recorded between 0.5 and 70 Hz frequency ranges. To ensure signal integrity, all channels were visually inspected for non-physiological artifacts. Segments containing muscle noise or amplifier saturation were excluded. The impedance of all electrodes was kept below 10 KΩ.

### 2.3. Preprocessing

Firstly, to suppress noise and unwanted frequency ranges, a 6th-order Infinite Impulse Response (IIR) Butterworth bandpass filter in the 0.5–64 Hz range was applied to the signal. A 50 Hz notch filter was then applied to remove 50 Hz noise. To remove eye movement artifacts, regression-based filtering (Equation (1)) was applied to the *EEG* signals using *EOG* signals. All processes were performed in MATLAB^®^ R2021b. All signal-processing steps were applied to both the EC and EO datasets [20].

(1) $${EEG}_{cor}={EEG}_{raw}-\gamma {EOG}_{left}-\delta {EOG}_{right}$$

where *γ* and *δ* are transmission coefficients between *EEG* and *EOG* signals.

### 2.4. Data Slicing

Due to the limited number of participants, a data-slicing strategy was adopted to increase the adequate sample size while strictly preventing information leakage. To this end, participant-level separation was enforced throughout the entire analysis pipeline. We employed a 5-fold nested cross-validation (CV) framework. In the outer loop, participants were split into five folds, with all data from a given participant assigned to a single fold. In each iteration, one fold containing complete participants was held out as the outer test set, while the remaining four folds formed the outer training set. This design ensured that no information from test participants was used during model selection or training. Within each outer training set, an inner cross-validation loop was used exclusively for model selection and optimization. Importantly, all signal segmentation was performed after the participant-level split. Specifically, each 5 min EO and EC resting-state EEG recording in the outer training data was divided into 10 non-overlapping 30 s segments, preserving the original label of the parent recording. All segments originating from the same participant were kept together and were never distributed across different folds [15,21,22]. Crucially, all feature-related procedures were confined to the outer training data only, including:

(i) fitting the Neighborhood Component Analysis (NCA) model,
(ii) selecting between EO and EC feature sets, and
(iii) optimizing the NCA feature-weight threshold within the inner CV loop.

**Figure 2:**
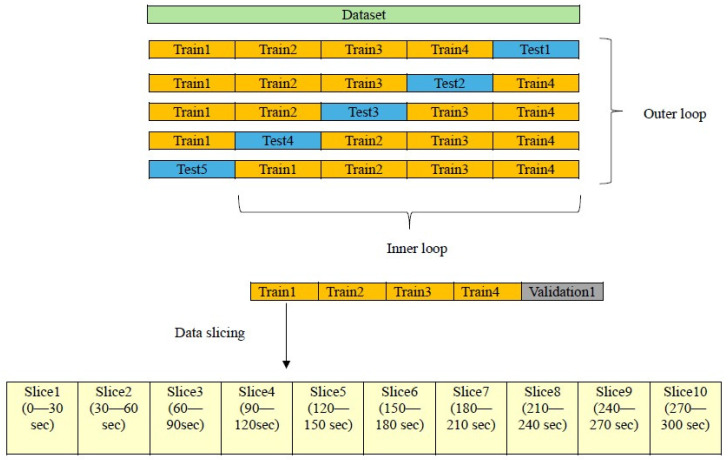
Scheme of Data splitting and participant-level slicing.

At no point was the outer test fold accessed during feature selection, threshold tuning, or model optimization (see Figure 2).

After completing the inner-loop optimization, the final NCA model and downstream classifiers and regressors were refit using only the complete outer training data. Subsequently, they evaluated the held-out participants in the outer test fold. This procedure was applied consistently across both classification and regression tasks.

Overall, this participant-level nested CV and data-slicing strategy ensured leakage-free evaluation and unbiased performance estimation, while enabling a comprehensive analysis in the absence of publicly available EEG datasets containing both BDI and MOCA scores.

### 2.5. Feature Extraction

In this study, we followed functional connectivity analysis approaches to extract features from EEG signals. Firstly, all segmented signals were divided into 5 EEG sub-bands: delta (0.5–4 Hz), theta (4–8 Hz), alpha (8–13 Hz), beta (13–30 Hz), and gamma (30–64 Hz) using a 6th-order IIR Butterworth bandpass filter. Coherence was used to quantify the spectral relationship between two EEG channels and is calculated from the spectral and cross-spectral densities of both channels, as shown in Equation (2).

(2) $$C_{x,y}\left(f\right)=\frac{{\left|G_{x,y}(f)\right|}^{2}}{G_{x,x}(f)\;G_{y,y}(f)}$$

In Equation (2), f represents the frequency range for each sub-band, *G*(*x,y*) (*f*) represents the cross-spectral density between two electrode channels, and *G*(*x,x*) (*f*), *G*(*y,y*) (*f*) represent auto spectral density for *x,* and *y*, where *x*, and *y* represent two different EEG channels. *C*(*x,y*) (*f*) represents the coherence value between two EEG electrodes and varies between 0 and 1 [22]. To estimate the spectral density, we used the Welch method with a 5 s Hamming window and 50% overlap. The Fast Fourier Transform (FFT) length was also set to the window length. To obtain the coherence value for each sub-band, we averaged individual bins in related sub-bands.

Another functional connectivity approach was the Phase Lock Index (PLI) [23]. PLI was used to quantify the synchronization of the inference phase between two EEG channels. For each EEG pair, PLI was calculated by Equation (3).

(3) $${PLI}_{x,y}=\left|\langle sign[\emptyset_{x}(t)-\emptyset_{y}(t)]\rangle \right|$$

The PLI values also range from 0 to 1. 0 indicates no connectivity between the two electrodes, and 1 means maximum connectivity. $\emptyset_{x}(t)$, and $\emptyset_{y}(t)$ represent a sudden phase of the signals that were recorded by the *x* and *y* channels. These phases were calculated by Equation (4).

(4) $$\emptyset_{x}\left(t\right)={tan}^{-1}(\frac{\hat{x\left(t\right)}}{x\left(t\right)})$$

where *x*(*t*) is the fundamental part of the signal and $\hat{x\left(t\right)}$ The complex part of the signal is calculated using the Hilbert transform. In this study, the Hilbert transform was computed using a 200-point FFT.

Granger Causality (GC) is an approach for quantifying causal relationships between EEG channels. This method is based on two basic principles: (i) the cause precedes the effect, and (ii) the cause significantly improves the prediction of the effect. GC occurs when the current values of *y*(*t*) can be better predicted using the past values of *x*(*t*). To compute GC between EEG channels, we employed univariate and bivariate vector autoregressive (V-AR) models implemented in MATLAB using the BSMART toolbox (2008) [24]. An AR model order of *p* = 10 was selected empirically, in accordance with previous studies and based on preliminary Akaike Information Criterion (AIC) testing.

The number of paths between EEG electrodes that are used in FC is calculated by Equation (5).

(5) $$Number\;of\;paths=\frac{N*\left(N-1\right)}{2}$$

In the connectivity-based feature extraction approach, the number of features is significant due to Equation (5). In this study, all features were extracted for all possible pairs of 16 electrodes (120 paths). All feature extraction processes were performed in MATLAB^®^ R2021b.

### 2.6. Feature Selection

As we mentioned before, all features were extracted for 5 different EEG sub-bands and all possible EEG electrode pathways (120 paths). It means that for each feature we have 600 (120 × 5), and for all three features it equals 1800 (120 × 5 × 3). To reduce the number of features and select the most important ones, we used the Neighborhood Component Analysis (NCA) method. The NCA is a feature selection method based on feature weighting to select the most discriminative features. We choose a threshold for the NCA method to select the most essential features. We applied the feature selection step in both classification and prediction tasks. The threshold values chosen in the feature selection method were determined experimentally for each task. The feature importance and weight values corresponding to the number of features at which the performance of machine learning algorithms begins to decrease were selected as thresholds. The feature weight thresholds were set to 0.5, 0.45, and 0.47 for classification, severity, and MOCA score prediction tasks, respectively. Feature weight thresholds were determined by systematically evaluating values in the range of 0.20–0.80 within the inner cross-validation folds and selecting the threshold that yielded the best cross-validated performance for each task. Feature selection methods were applied only to feature (EC or EO) sets that show better performance in both classification and regression tasks.

### 2.7. Machine Learning Based Classification

To classify groups into DD patients and healthy control subjects, we used the support vector machine (SVM) with a linear kernel, K-nearest neighbor (KNN) for k = 9 and 11 values, Euclidean distance measurement, a discriminant analysis-based ensemble learning algorithm with the Bootstrap Aggregating (Bagging) method, and a Neural Network (ANN). The ANN architecture consisted of three fully connected layers with 20, 10, and 5 neurons, respectively. To limit model complexity and mitigate overfitting risk, given the small number of unique participants, the network was intentionally kept shallow. The sigmoid activation function was applied only in the output layer to model the final prediction stage, while no nonlinear activation functions were used in the hidden layers. This design allowed the model to learn hierarchical linear combinations of EEG connectivity features without introducing strong nonlinearities that could lead to unstable estimates in small-sample settings. The learning rate was fixed at 0.01 based on stable convergence observed during preliminary experiments. The number of neurons per layer was selected empirically and not optimized through extensive hyperparameter tuning within the inner cross-validation folds, reflecting the exploratory, proof-of-concept nature of the study. This step was repeated for each input (EC/EO) separately. In the classification task, we measure classifier performance using accuracy, sensitivity (recall), specificity, F1-score (Equation (6)), and the Matthews Correlation Coefficient (MCC) (Equation (7)). MCC values range from −1 to 1. Where MCC = −1 represents that there is no agreement between predicted and actual labels, MCC = 0 represents a random guess, and MCC = 1 means that there is a complete agreement between predicted and actual labels, and the area under the curve (AUC). All reported performance metrics were computed on the held-out test data of the outer cross-validation loop.

(6) $$f1-score=2\times \frac{Sensitivity\times Precision}{Sensitivity+Precision}$$

(7) $$MCC=\frac{TP\times TN-FP\times FN}{\sqrt{\left(TP+FP)(TP+FN)(TN+FP)(TN+FN\right)}}$$

### 2.8. Machine Learning Based Regression

To predict the severity of DD patients according to the BDI score and their cognitive impairment score according to the MOCA score, we utilized the Support Vector Regressor (SVR) with a linear kernel, an ensemble learning regressor model based on a decision tree with the Bagging method, and Artificial Neural Network Regressor (ANNR) with three fully connected layers with sizes of 20, 10, and 5 and a sigmoid activation function. This step was repeated for each input (EC/EO) separately. We used R-squared (*r*^2^) and MSE as performance metrics in the prediction task. All reported performance metrics were computed on the held-out test data of the outer cross-validation loop.

(8) $$r^{2}=1-\sum_{i=1}^{n}({\frac{y_{i}-{\hat{y}}_{i}}{y_{i}-\bar{y}})}^{2}$$

(9) $$MSE=\frac{1}{n}\sum_{i=1}^{n}({y_{i}-{\hat{y}}_{i})}^{2}$$

where n represents the number of observations, $y_{i}$ represents the actual value, ${\hat{y}}_{i}\;$ represents the predicted value, and $\bar{y}\;$ represents the mean of the actual value [19]. For the regression analyses predicting BDI and MOCA scores, the same participant-level 5-fold nested cross-validation strategy described in Section 2.4 was applied. All EEG segments derived from a given participant were always assigned to the same fold to ensure strict subject-level separation. Within each outer cross-validation iteration, an inner cross-validation loop was used exclusively for model selection and optimization. This included fitting the NCA model, optimizing the feature-weight threshold, and selecting between the EO and EC feature sets, all using only the outer training data. After completing inner-loop optimization, the final regression models were refit using the complete outer-training set and evaluated on the held-out participants in the outer test fold.

### 2.9. Statistical Analysis

To investigate the possibility of selected features that the NCA chosen as a biomarker, we used a statistical *t*-test (*p* < 0.05) for selected features in the classification task to evaluate whether the differences in the selected features were statistically meaningful and Pearson’s correlation test (*p* < 0.05) in the regression task to determine the linear relationship between selected features and the target variable. We applied these tests only to the original data set and only to feature subsets selected by the NCA method. Feature-wise statistical analyses, including independent-samples *t*-tests and Pearson correlation analyses, were performed to support the interpretation of EEG features identified by the NCA. For this reason, all statistical tests in the present study were conducted in an exploratory and descriptive manner rather than as confirmatory inferential analyses. Importantly, the primary evidence for feature relevance and model performance is provided by the participant-level nested cross-validated machine learning framework. At the same time, statistical analyses provide complementary insight into potential neurophysiological patterns and their associations with clinical measures.

## 3. Results

### 3.1. Classification Results

**Figure 3:**
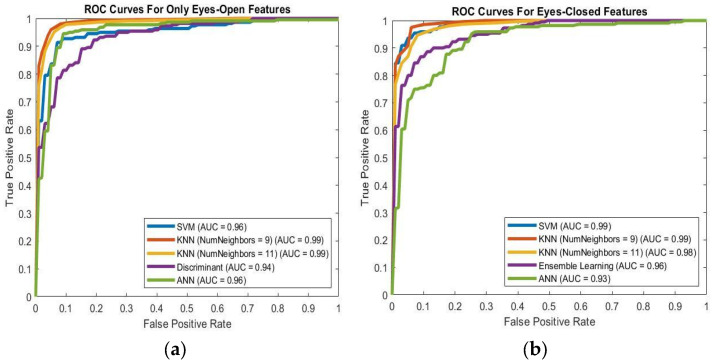
(**a**) AUC results for EO features in the control/depression classification task. (**b**) AUC results for EC features in the control/depression classification task. (AUC: Area Under Curve).

In Table 2 and Figure 3a,b, the average performances of all classifiers are shown for features extracted from EO and EC EEG signals. The KNN (K = 9) classifier performed better than other algorithms in the DD/healthy control classification task when EO features were used as inputs of the classifiers. In contrast, the SVM classifier achieved the highest performance in the DD/healthy control classification task when EC features were used as classifier inputs. According to the results in Table 2 and Figure 3a,b, the extracted features from EC EEG signals perform better than those from EO EEG signals. As a result, we applied feature selection methods to identify optimal features and pathways for EC EEG signals.

**Figure 4:**
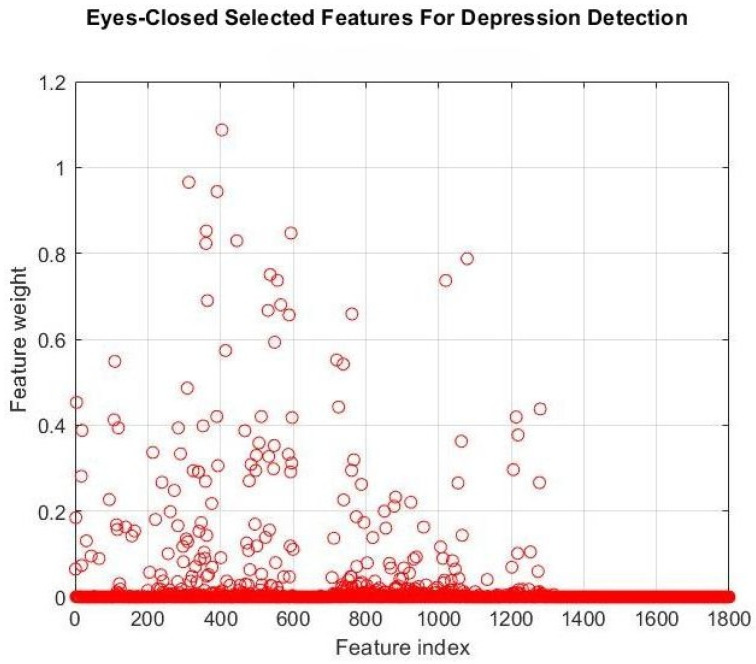
The importance of each EC feature in the control/depression classification task.

**Figure 5:**
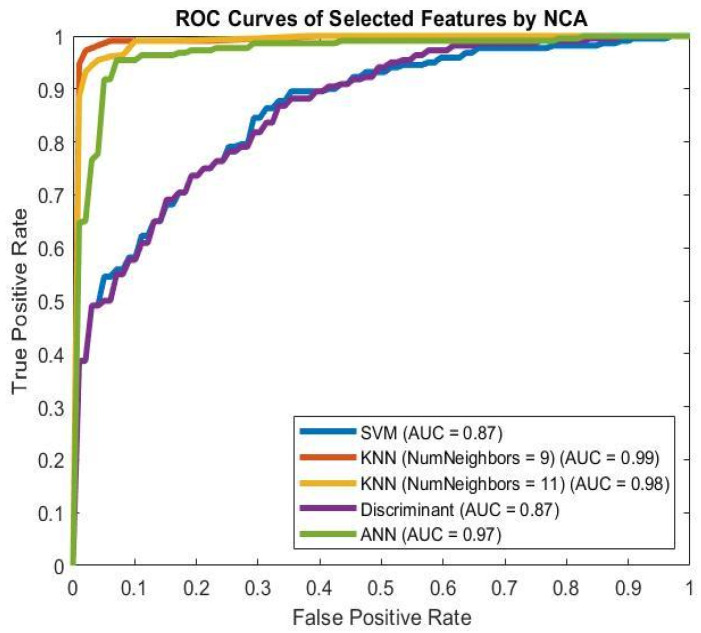
AUC results for selected EC features by the NCA method in the control/depression classification task. (AUC: Area Under Curve).

To reduce the number of features and identify the optimal subset from 1800 EC features, we applied the NCA method with a 0.5 threshold for feature weights. The number of features was reduced to 21. Figure 4 shows the importance of each EC feature to classifier performance. Table 3 and Figure 5 show the performance of the classifiers when only the 21 selected features were used as inputs. The KNN (K = 9) classifier achieves the best performance in classifying DD patients and healthy control subjects.

To investigate potential biomarkers for the classification task, a *t*-test was applied to the feature sets that performed best at distinguishing DD patients from healthy control subjects.

Table 4 presents the selected features from the NCA method and the *t*-test results for these values between DD patients and healthy control groups. According to Table 4, coherence and PLI features from frontal and temporal-related pathways, as well as the alpha, beta, and gamma sub-bands, show significant differences between the DD and healthy control groups.

### 3.2. Prediction Results

In this section, the prediction results of depression severity and MOCA score were reported.

Table 5 presents the performance of ML algorithms in predicting the severity of depression. The ENR algorithm achieved a mean *r*^2^ of 0.88 ± 0.04 and an average MSE of 10.76 ± 19.42 when EO features were used. This regressor also yielded a mean *r*^2^ of 0.88 ± 0.01 and an average MSE of 9.01 ± 16.20 when EC features were used. According to this table, EC features performed better than EO features; therefore, the feature selection approach was applied to EC features.

**Figure 6:**
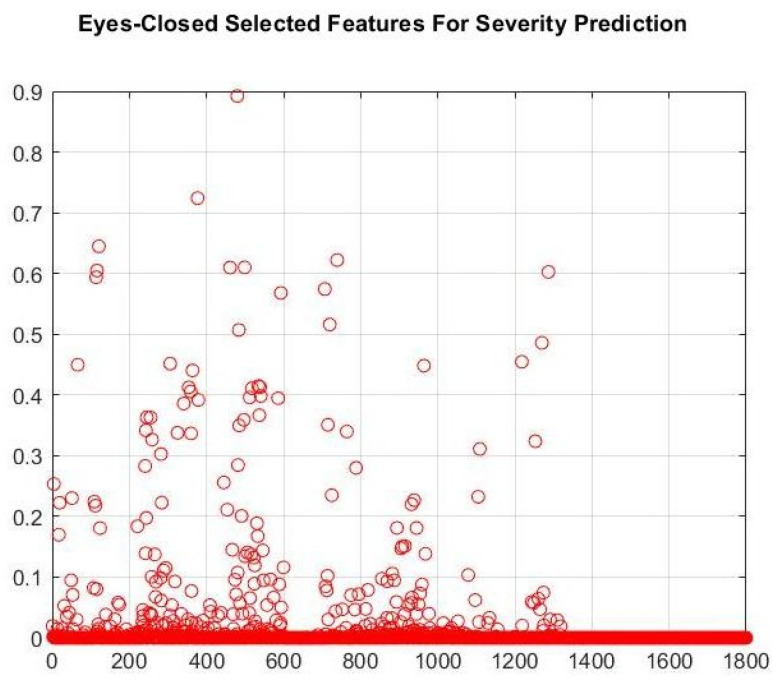
The importance of each EC feature in the DD severity prediction task.

Table 6 presents the performance of ML algorithms when NCA’s selected features were used as inputs. As shown in Figure 6, the number of features in this approach was reduced to 20 when we applied the NCA method with a 0.45 threshold for feature weight. According to Table 6, the ANNR regression algorithm performed better when 20 selected features from the NCA method were used as inputs to the regressor algorithms in the DD-patients’ severity-prediction task.

To investigate potential biomarkers for predicting the severity of DD patients, Pearson’s correlation test was applied to selected features using the NCA method. Table 7 presents the Pearson correlation coefficients between the features chosen by the NCA method and the BDI scores, which indicate the severity of depression. As listed in Table 7. Coherence and PLI features, which generally arise from frontal and temporal-related paths and the theta, alpha, beta, and gamma sub-bands, are positively correlated and significantly meaningful (*p* < 0.05) with BDI scores that represent DD severity.

Table 8 presents the performance of ML algorithms in predicting the MOCA score. The ENR algorithm achieved a mean *r*^2^ of 0.86 ± 0.04 and an average MSE of 0.92 ± 1.75 when EO features were used. In contrast, the ANR algorithm achieved the best performance, with a mean *r*^2^ of 0.88 ± 0.10 and an average MSE of 0.33 ± 0.83, when EC features were used. According to this table, EC features performed better than EO features; therefore, a feature selection approach was applied to EC features.

**Figure 7:**
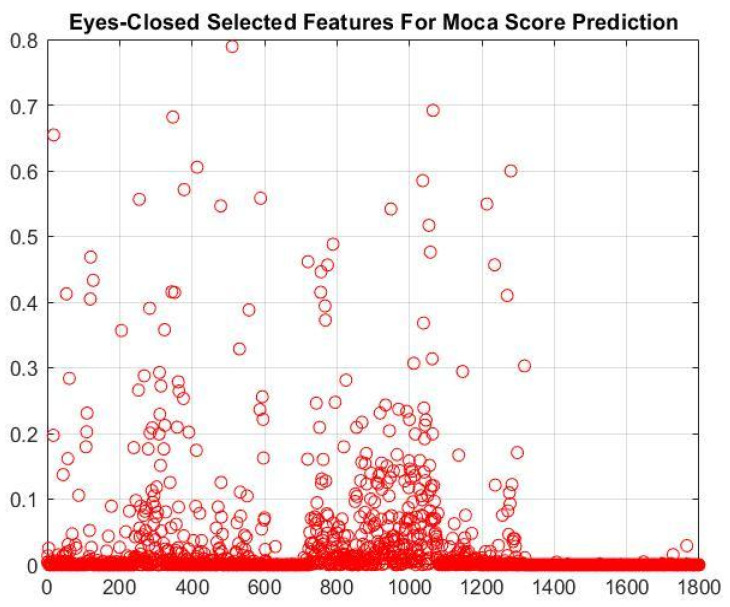
The importance of each EC feature in the DD patients’ MOCA score task.

Table 9 presents the performance of ML algorithms when the NCA-selected features were used as inputs. As shown in Figure 7, the NCA method reduced the feature set to 17 when a 0.47 feature-weight threshold was applied. The NCA-selected features show the best performance in predicting the MOCA score. The ANNR regression algorithm performed better when 17 selected features by the NCA method were used as inputs of the regressor algorithms in the DD patients’ MOCA score prediction task.

To investigate potential biomarkers for predicting MOCA scores in DD patients, Pearson’s correlation test was applied to selected features identified by the NCA method. Table 10. represent the Pearson’s correlation results of features chosen by the NCA method with the MOCA scores. As listed in Table 10, apart from “T5-P3, alpha, coherence”, “T3-O1, beta, PLI”, and “T4-O1, beta, PLI” features, coherence, and PLI features that generally came from frontal and temporal-related paths and theta, alpha, beta, and gamma sub-bands are positively correlated and significantly meaningful (*p* < 0.05) with the MOCA scores that represent DD patients’ cognitive impairment.

## 4. Discussion

Automatic diagnosis, disease severity, and cognitive impairment level of DD patients using machine learning and investigating potential biomarkers are emerging fields in psychiatry. As previously addressed, general approaches to identifying DD and assessing its severity, including cognitive impairment level, are based on clinical examination of DD patients, family history, and self-reported questionnaires. All these methods are time-consuming, subjective, and not repeatable. Additionally, the lack of well-experienced professionals is an obstacle to detecting DD at an early stage. For these reasons, we proposed a connectivity-based framework to detect DD and assess its severity relative to cognitive impairment level, by introducing optimal features and pathways using EEG and machine learning methods. Based on our review of the literature, this study is the first to investigate the potential of connectivity-based features as biomarkers for detecting DD patients, predicting DD severity and cognitive impairment scores, and evaluating their potential as biomarkers.

In the patient/control classification task, we found the best performance among all models as 97.66% ± 2.05% accuracy, 99.20% ± 1.10% sensitivity, 95.91% ± 4.66% specificity, 98.00% ± 1.02% f1-Score, and 0.95 ± 0.48 MCC rate by using 21 EC features selected by NCA method as inputs of the KNN (K = 9) classifier. In the DD severity prediction task, we found the best *r*^2^ value of 0.89 ± 0.10 and the minimum MSE of 3.96 ± 17.05 using 20 EC features selected by the NCA method as inputs to the ANN regressor (Table 1). The standard deviation of BDI scores for DD patients is 9.14. It may lead to a high standard deviation in the MSE result of severity prediction. In the DD patients’ MOCA scores prediction task, we achieved the maximum average *r*^2^ value of 0.89 ± 0.06 and the minimum MSE value of 0.23 ± 0.45 when using 17 selected EC features by the NCA method as inputs to the ANN regressor.

In this study, we also compared the performance of different FC EEG features extracted from EO and EC resting-state EEG signals for the detection of depression and for predicting the patient’s severity and cognitive impairment score. Our key findings can be summarized as follows: (1) FC features extracted from EC resting-state EEG signals performed better than EO features across all tasks. (2) The selected features by the NCA method showed better performance than other selected features by other feature selection methods and all EC features. (3) The optimal feature subsets selected by the NCA method generally were from the alpha, beta, and gamma sub-bands and frontal, temporal-related pathways that were extracted by coherence and PLI methods.

### 4.1. Efficiency of Feature Selection Approaches in Machine Learning Algorithms’ Performance

According to Table 2 and Figure 3a,b, EC features (1800 features) showed better performance compared to EO features (1800 features) in the DD/Healthy Control classification. Although the highest mean values in Table 2 are highlighted to facilitate comparison, the differences between the best and second-best classifiers are not always clearly distinguishable when the associated standard deviations are taken into account. This suggests that multiple models exhibit similar performance levels rather than one model consistently outperforming the others. For this reason, our focus was on identifying the most stable algorithm, defined by a combination of high average performance and low variability across cross-validation folds, as this pattern reflects more reliable, robust behavior. Importantly, this interpretation is also supported by the MCC scores, which provide greater separation between classifiers even when other performance metrics are closely clustered. After applying the NCA-selected features approach to EC features, the number of features was reduced to 21 (see Figure 4). According to Table 3 and Figure 4, the 21 selected EC features by the NCA method showed better performance than other feature sets. The relatively large standard deviation in the MCC values indicates notable variability across cross-validation folds, as expected when participant-level validation is performed on a limited number of subjects with heterogeneous clinical characteristics. Although MCC is intrinsically bounded between −1 and 1 at the level of individual folds, the use of mean ± standard deviation as a summary statistic may mathematically extend beyond these bounds without implying that any fold produced invalid values. Instead, this pattern reflects the sensitivity of performance estimates to the specific composition of participants in each fold, highlighting subject-level heterogeneity.

In the DD severity prediction task, according to Table 5, EC features showed better performance than EO features. As a result, the feature selection approach was applied to the EC features (1800 features). The NCA method reduced the feature set to 20 (see Figure 6). The 20 selected features showed better performance than other feature sets.

To predict the cognitive impairment score of DD patients assessed with the MOCA test, we also used EO and EC features. According to Table 9, EC features performed better than EO features, so the feature selection approach was applied to EC features (1800 features). The NCA method reduced the feature set to 17 (see Figure 7). The 17 selected features outperformed other feature sets.

The regression analyses to predict BDI and MOCA scores were conducted using data from the depressed patient group only, which comprised a relatively small number of unique participants (*n* = 22). Although we carefully applied participant-level nested cross-validation to minimize data leakage and reduce overfitting, the limited sample size inevitably constrains the generalizability of the regression models, particularly those based on neural networks. For this reason, the regression results should be viewed primarily as exploratory, proof-of-concept findings that illustrate the potential of EEG functional connectivity features for estimating symptom severity and cognitive variability in depression, rather than as finalized clinical prediction models.

EC-related features performed better than EO-related features across all tasks. This disparity may be attributed to the EC position diminishing external sensory input, hence facilitating a greater focus on intrinsic brain activities [25]. In this condition, the brain’s functional connection patterns increasingly mirror its internal regulatory processes, possibly revealing disturbances in top-down regulation mechanisms. Top-down regulatory mechanisms denote the brain’s capacity to regulate sensory input, emotional reactions, and cognitive functions. In depressive disorders, these top-down pathways are frequently compromised, leading to dysregulated communication between higher-order areas, such as the prefrontal cortex, and lower-order regions involved in sensory and affective processing, including subcortical structures and sensory cortices. The eyes-closed state reduces outward sensory interference, hence enhancing the perception of these internal disruptions. The EC state may elucidate the altered neural networks characteristic of sadness by amplifying the brain’s regulatory deficiencies. This increased sensitivity to alterations in functional connectivity provides significant insights into the impact of depression on the brain’s processing of external stimuli and its internal regulatory capabilities [26,27,28].

According to Table 4, Table 7 and Table 10, no GC features are selected by the NCA method. While GC was considered a directional functional connectivity measure at the feature-extraction stage, it was not chosen among the NCA-selected features. This result is likely due to assumptions in standard GC analysis, such as reasonably steady signals, a linear autoregressive model structure, and sufficient sampling quality of all relevant neural sources. Nevertheless, EEG signals are non-stationary, noisy, and nonlinear, and are subject to volume conduction, hidden sources, and residual artifacts. If these assumptions are violated, false causal interactions and biased GC estimates may result, especially in small-sample resting-state EEG studies.

### 4.2. Investigate Potential Biomarkers

This study aimed to investigate the potential of the selected features as biomarkers. We applied a *t*-test on the selected features using the NCA method in the classification task. Table 4 lists all features selected by the NCA method. Among these features, those that show significant differences (*p* < 0.05) after applying the *t*-test between DD patients and healthy subject controls might be considered as biomarkers. Table 4 shows features that show significant differences between the two groups, which primarily originate in the frontal and temporal regions. This outcome aligns with the literature suggesting that FC in frontal and temporal regions may be related to deficits in cognitive processing, such as memory and emotion, in DD patients [16,29]. The presence of features extracted from alpha, beta, and gamma sub-bands aligns with other studies. The alpha band plays a core role in inhibitory processing and is related to deficits in cognitive and emotional control in DD patients [30,31,32]. In a previous study, it was reported that the beta band was found to be higher in DD patients and associated with the number of depressive episodes [29]. The high number of beta-related features among the biomarkers supports the hypothesis that claimed synchronization of the beta band is related to a cognitive idling rhythm present in DD patients [33,34]. Gamma-band FC abnormalities have been reported in DD patients [35,36] and may be related to signaling between serotonin and γ-aminobutyric acid (GABA) [37]; this band is also sensitive to emotion processing.

In addition to the mid- and high-frequency oscillation-based features selected as biomarkers, low-frequency oscillation-based features were also selected. This finding can be explained by studies that claimed state-dependent alterations in delta and theta sub-bands FC [38,39]. Both PLI and coherence abnormalities in the frontal and frontotemporal pathways have remained longitudinally associated with the neurophysiological manifestations of DD. These measures reveal the nature of the non-dominant quantitative representation of disrupted psychomotor communication circuitry, providing DD-focused PLI-coherence integration targets for directional neuromodulation to reinstate disrupted neural synchrony. PLI captures phase-shifted synchrony in brain oscillations by signal-processing methods that reduce volume-conduction bias and reveal frequency-dependent disturbances in large cognitive and emotional control networks. The dorsolateral prefrontal cortex focally modulates PLI and frontal-vagal circuits [40,41]. Similarly, coherence studies have shown disturbances in synchrony, primarily manifest as residual hyper- and hypo-coherence, within and between fronto-parietal circuits associated with depressive cognitive dysfunction, attention, and motivation [42,43,44].

According to Table 7, it is noteworthy that among the features selected by the NCA method, those significantly correlated with patients’ BDI scores are from the frontal and temporal regions and the theta, alpha, beta, and gamma sub-bands. The PLI, as well as disturbances of coherence within the subject with depression, shows the way symptom severity may shape abnormal brain connectivity. Differences in coherence and PLI across multiple levels of depression, specifically in the frontal and frontotemporal regions, might be indicative of the cognitive and emotional dysregulation patterns characteristic of depressive disorders. Deficits in cognitive abilities such as attention, learning, memory, and emotion regulation characterize depression [45,46]. In depression, the alpha frequency bands show altered activity patterns that reflect the dysregulated functional connectivity and are related to depression severity [47]. Beta oscillations are associated with active thinking, attention, and motor activity [48]. Theta waves are linked to memory, emotional processing, and information content-specific coding levels during response inhibition [49]. Altered gamma activity in depressed individuals could reflect deficits in higher-order cognitive processes and attention regulation [48].

Table 10 shows that, among the features selected by the NCA method, those from the frontal and temporal pathways, as well as the delta, alpha, and beta sub-bands, are significantly correlated with patients’ MOCA scores. These results showed that altered functional connectivity of EEG activity in depression highlights the disrupted communication between brain regions that regulate mood, cognition, and emotional responses. These frequency bands and brain regions play crucial roles in reflecting these abnormalities and can provide valuable biomarkers for understanding depression’s neurophysiological underpinnings and cognitive impairment [50]. The negative correlation between the MOCA score and the coherence of the beta sub-band aligns with Roh and colleagues’ findings of a negative correlation between beta power and the inattention score in DD patients [48]. Although the mean MOCA score was slightly lower in the depressed group than in the healthy controls (as shown in Table 1), both groups scored at or above the commonly used cutoff for global cognitive impairment. This result should therefore be interpreted with caution. The MOCA is primarily a brief screening instrument designed to identify global cognitive impairment, particularly in conditions such as mild cognitive impairment and dementia, and it may be less sensitive to subtle or domain-specific cognitive changes. In the present study, the patient group primarily consisted of individuals experiencing a first depressive episode and who had not yet received pharmacological treatment, a context in which preserved performance on global cognitive screening measures is not unexpected. At early stages of depression, cognitive alterations are often selective, involving domains such as executive functioning, attention, or processing speed, which may not be fully reflected in the total MOCA score. Accordingly, the relatively similar MOCA scores observed between groups are more likely to reflect the limitations of global screening tools and a potential ceiling effect rather than an absence of cognitive involvement in depressive disorder. A key limitation of the present study is the relatively small number of unique participants, which inevitably affects the stability and generalizability of the reported results. Although participant-level nested cross-validation and conservative modeling choices were employed to mitigate overfitting, performance estimates, particularly for the regression analyses, may still be sensitive to the specific composition of subjects across folds. Larger, more diverse datasets are therefore required to obtain more stable, robust performance estimates and to assess the generalizability of the proposed framework fully.

Nevertheless, despite this limitation, the findings of the present study remain meaningful. The consistent identification of specific EEG functional connectivity patterns across classification and regression tasks suggests that the proposed approach captures relevant neurophysiological information related to depressive symptom severity and cognitive vulnerability. As such, the results should be viewed as an essential proof of concept, demonstrating the potential of EEG-based functional connectivity features within a carefully controlled validation framework, and motivating future studies with larger cohorts to validate further and extend these findings.

The identified biomarkers, derived from NCA-selected features across all tasks for coherence, PLI, and Granger causality metrics, consistently highlight functional disruptions in frontal-temporal connectivity and oscillatory dynamics in DD patients. Importantly, these findings provide not only theoretical insights but also actionable clinical relevance: the altered EEG signatures in frontal theta, alpha, and beta bands correlated with both depression severity and cognitive impairment, and can potentially be used as objective indicators to complement traditional psychiatric evaluations. Such biomarkers could assist clinicians in identifying patients at higher risk for cognitive decline or treatment-resistant depression and may help in monitoring treatment response over time. The integration of these interpretable biomarkers into EEG-based decision-support tools holds promise for more personalized, precision-guided interventions in psychiatric care.

### 4.3. Comparison with Other Studies

Many studies in the literature propose methods for detecting and classifying DD patients. These studies used EEG-based time-domain, frequency-domain, linear, nonlinear, and FC features. According to Equation (5), FC approaches increase the feature vector size. We used several feature selection methods to identify optimal EEG sub-bands, pathways, and features that are more relevant for classifying DD patients/healthy control subjects. Earl and colleagues used coherence-based FC features from EEG signals recorded during the resting, happy, and sad states. Using the Gini criterion as a feature selector and a random forest classifier to detect depression and enhancement, they reported a 60% classification accuracy rate with 93 resting-state FC features. They reached 80% maximum accuracy using 84 selected happy-state features [16]. Peng et al. used the PLI method on EC resting-state brain activity from DD patients and healthy control subjects. They reported 92.73% classification using an SVM classifier and the Kendall rank correlation coefficient as the feature selection method. They reached this rate using 249 features extracted from whole EEG signals, without separating them into sub-bands [51]. Chen et al. also conducted a study to diagnose primary DD using the PLI and phase-locking value (PLV) methods. They achieved 88.73% accuracy, 90.67% sensitivity, and 86% specificity using 29 features selected by a PCA, K-means, and mutual information (PKM) method as inputs to a CNN classifier [17]. Xia and colleagues [52] proposed an end-to-end deep learning model to classify patients with depression by extracting coherence, PLI, and transfer entropy features across the full frequency range of EEG. They used a CNN classifier, achieving an average accuracy of 91.06%. In the current study, we reached the maximum average of 97.66% ± 2.05% accuracy, 99.20% ± 1.10% sensitivity, 95.91% ± 4.66% specificity, 98.00% ± 1.02% f1-Score, and 0.95 ± 0.48 MCC rate when we used FC based 21 selected EC features by the NCA method as inputs of the KNN (K = 9) classifier. Table 11 summarizes the literature review.

**Table 11:** Comparison of other connectivity-based classification studies.

Study	Subjects	Features	Features Selection	Number of Selected Features	Classifier	Results
Earl et al.	24 DD 25 HC	PLI	Gini	23	Random Forest	Accuracy = 92.3% Precision = 60% Sensitivity = 60%
Peng et al.	27 DD 28 HC	PLI	Altered Kendall Rank Correlation	249	SVM	Accuracy = 92.73%
Chen et al.	34 DD 30 HC	PLI, PLV, Coh, Icoh, MI, PCC, WPLI	PKM, PKC	8	CNN	Accuracy = 88.73% Sensitivity = 90.67% Specificity = 86%
Xia et al.	34 DD 30 HC	PLV, Coh, TE	N. A	N. A	CNN	Accuracy = 91.06% Sensitivity = 91.33% Specificity = 89.68%
Current Study	22 DD 25 HC	Coh, PLI, Granger	NCA	21	KNN (K = 9)	Accuracy = 97.66% Sensitivity = 99.20% Specificity = 95.91% f1-score = 98% MCC = 0.95

Coh: Coherence, PLI: Phase lag index, PLV: Phase-locked value, Icoh: Imaginary coherence, MI: Mutual information, WPLI: Weighted phase lag index, PCC: Pearson correlation coefficient, TE: Transfer entropy.

In our literature review, we identified only two studies that attempted to predict DD severity based on BDI scores using EEG and artificial intelligence algorithms. Hashempour et al. conducted a survey to predict BDI scores in DD patients. They used raw EEG signals from DD patients and a CNN to predict patients’ BDI scores. They reported MSE of 5.64 ± 1.6 and MAE of 1.73 ± 0.27 when using EO EEG signals [19]. Kang et al. also conducted a study to predict DD severity from BDI scores using power analysis of EEG signals and a CNN regressor. They reported a 3.46 RMSE for the prediction task [18]. Compared to these studies, our approach showed notably higher performance, with an *r*^2^ value of 0.89 ± 0.10 and an MSE of 3.96 ± 17.05, when we used FC-based 20 selected EC features by the NCA method as inputs to the ANN regressor. To the best of the researchers’ knowledge, this is the first study to propose an algorithm using EEG connectivity-based features and a machine learning algorithm, investigating optimal feature types, EEG sub-bands, and electrode paths.

None of the previous studies attempted to predict cognitive impairment scores in DD patients based on MOCA scores using EEG signals. As a result, we cannot compare our results with literature. As mentioned above, we obtained the maximum average *r*^2^ value of 0.89 ± 0.06 and the minimum MSE value of 0.23 ± 0.45 when we used FC-based 17 selected EC features by the NCA method as inputs to the ANN regressor.

To the best of our knowledge, this is the first study to present an interpretable and comprehensive EEG-based machine learning framework that jointly addresses depression diagnosis, severity estimation, and cognitive impairment prediction. It offers not only state-of-the-art performance but also clinically actionable insights, thereby providing a valuable contribution to both the scientific literature and potential real-world psychiatric applications.

## 5. Conclusions

In this study, we proposed a connectivity-based machine learning framework that leverages resting-state EEG functional connectivity features to support risk-oriented screening and decision support in depressive disorder (DD), rather than establishing a clinical diagnosis. Using optimized feature selection, we aimed to identify the most informative connectivity pathways and to model symptom severity and cognitive vulnerability in individuals clinically diagnosed with DD. Our findings suggest that eyes-closed resting-state EEG functional connectivity measures, particularly coherence and PLI features derived from alpha, beta, and gamma sub-bands, provide meaningful neurophysiological information relevant to depressive risk assessment. Connectivity patterns within frontal and frontal–temporal pathways consistently emerged as informative features across both discrimination and regression analyses, highlighting their potential relevance for understanding neurophysiological alterations associated with depression. The proposed framework offers an objective, data-driven approach that may complement clinical evaluation by supporting early risk identification and contributing to personalized treatment planning, rather than replacing standard diagnostic procedures. The interpretability of the selected EEG-based features further enhances the framework’s potential clinical value, as these biomarkers can be integrated into decision-support tools to monitor symptom severity, cognitive vulnerability, and treatment-related changes over time. Several limitations should be acknowledged. Although the sample size was sufficient for an exploratory analysis, it remains limited for drawing definitive conclusions about the broader DD population. In addition, the observed associations between EEG connectivity features and clinical measures such as BDI and MOCA scores require validation in larger, independent cohorts. The lack of publicly available EEG datasets that include both MOCA and BDI assessments currently restricts external validation and cross-dataset comparisons. Future work will focus on validating the proposed framework across larger, more diverse populations and on extending it to model individual responses to specific therapeutic interventions. Such efforts may further contribute to the development of EEG-based decision-support systems that enhance personalized psychiatric care while remaining aligned with established clinical practice.

## Figures and Tables

**Table 1:** The summary of demographic and test scores for the two groups.

Groups	Healthy Controls	DD Patients
Age	30.40 ± 6.65	28.04 ± 8.55
BDI Score	6.60 ± 3.70	28.68 ± 9.14
MOCA Score	28.04 ± 1.92	26.09 ± 2.02

**Table 2:** Performance values of classifiers in the control/depression classification task with EO and EC features (MCC: Matthew’s Correlation Coefficient). Note: Bold values indicate the best performing results, while underlined values indicate the second-best results.

		SVM	KNN (K = 9)	KNN (K = 11)	Ensemble Learning	ANN
EO	Accuracy (%)	92.77 ± 1.39	**94.89 ± 0.89**	91.91 ± 1.93	85.32 ± 1.90	92.34 ± 1.75
	Sensitivity (%)	94.00 ± 3.74	**98.00 ± 2.00**	98.80 ± 1.10	86.40 ± 4.34	87.30 ± 3.66
	Specificity (%)	91.36 ± 3.37	**91.36 ± 2.96**	84.09 ± 4.55	84.09 ± 2.27	91.82 ± 3.80
	F1-Score (%)	93.18 ± 3.23	**92.11 ± 5.32**	95.14 ± 4.04	89.71 ± 3.23	90.08 ± 2.11
	MCC	0.85 ± 0.06	**0.90 ± 0.05**	0.91 ± 0.08	0.70 ± 0.03	0.79 ± 0.06
EC	Accuracy (%)	**95.74 ± 2.26**	95.11 ± 2.67	91.70 ± 3.31	88.30 ± 2.26	94.89 ± 2.75
	Sensitivity (%)	**99.60 ± 0.89**	98.00 ± 2.00	97.20 ± 2.28	91.60 ± 5.90	92.08 ± 5.03
	Specificity (%)	**91.36 ± 4.37**	91.82 ± 4.13	85.45 ± 4.98	84.55 ± 3.73	95.00 ± 2.49
	F1-Score (%)	**95.13 ± 3.74**	95.58 ± 4.69	94.11 ± 1.72	92.56 ± 2.33	91.34 ± 8.23
	MCC	**0.93 ± 0.07**	0.90 ± 0.06	0.84 ± 0.13	0.76 ± 0.10	0.87 ± 0.13

**Table 3:** Performance values of classifiers in the control/depression classification task with EC selected features by the NCA method. (MCC: Matthew’s Correlation Coefficient). Note: Bold values indicate the best performing results, while underlined values indicate the second-best results.

		SVM	KNN (K = 9)	KNN (K = 11)	Ensemble Learning	ANN
NCA Selected Features from EC	Accuracy (%)	81.83 ± 4.91	**97.66 ± 2.05**	95.23 ± 2.21	81.62 ± 4.41	90.15 ± 2.67
	Sensitivity (%)	79.60 ± 12.12	**99.20 ± 1.10**	99.20 ± 1.10	78.40 ± 9.10	91.86 ± 4.34
	Specificity (%)	67.27 ± 5.70	**95.91 ± 4.66**	95.00 ± 5.18	68.18 ± 5.79	89.55 ± 2.03
	F1-Score (%)	83.17 ± 5.12	**94.42 ± 4.12**	91.45 ± 5.78	85.00 ± 8.63	91.47 ± 10.45
	MCC	0.47 ± 0.89	**0.95 ± 0.48**	0.95 ± 0.36	0.46 ± 0.71	0.81 ± 0.51

**Table 4:** *t*-test Results for EC selected features by the NCA method in the classification task.

Features Name (Pathways, Sub-Band, Type)	*t*-Test Result (*p* < 0.05)
T5-O1, Delta, Coherence	n.s *
F8-P4, Alpha, Coherence	n.s
O1-O2, Alpha, Coherence	*p* < 0.05
Fp1-Fp2, Beta, Coherence	*p* < 0.05
Fp1-F7, Beta, Coherence	*p* < 0.05
F3-F7, Beta, Coherence	*p* < 0.05
F4-F8, Beta, Coherence	*p* < 0.05
F4-O2, Beta, Coherence	n.s
C4-T3, Beta, Coherence	*p* < 0.05
F4-P3, Gamma, Coherence	n.s
F7-C4, Gamma, Coherence	*p* < 0.05
F8-T4, Gamma, Coherence	*p* < 0.05
C3-T3, Gamma, Coherence	*p* < 0.05
C4-T4, Gamma, Coherence	*p* < 0.05
T5-O1, Gamma, Coherence	n.s
T6-O2, Gamma, Coherence	*p* < 0.05
O1-O2, Delta, PLI	*p* < 0.05
Fp2-F7, Theta, PLI	*p* < 0.05
F3-O2, Theta, PLI	*p* < 0.05
F7-T5, Alpha, PLI	*p* < 0.05
P4-O2, Alpha, PLI	*p* < 0.05

* non-significant difference.

**Table 5:** Results of the prediction of depression severity of DD patients using EO and EC features. Note: Bold values indicate the best-performing results, while underlined values indicate the second-best results.

		SVR	ANNR	ENR
EO	*r* ^2^	0.29 ± 0.02	0.2 ± 0.34	**0.88 ± 0.04**
	MSE	59.32 ± 79.40	105.53 ± 158.17	**10.76 ± 19.42**
EC	*r* ^2^	0.37 ± 0.03	0.38 ± 0.25	**0.88 ± 0.01**
	MSE	54.29 ± 81.29	46.07 ± 105.89	**9.01 ± 16.2**

**Table 6:** Results of the prediction of the depression severity using selected EC features by the NCA method. Note: Bold values indicate the best performing results, while underlined values indicate the second-best results.

		SVR	ANNR	ENR
NCA Selected Features from EC	*r* ^2^	0.38 ± 0.04	**0.89 ± 0.10**	0.83 ± 0.04
	MSE	43.23 ± 79.67	**3.96 ± 17.05**	11.73 ± 17.66

**Table 7:** Correlation Results for EC-selected features by the NCA method in DD severity prediction Task.

Features Name (Pathways, Sub-Band, Type)	Correlation Coefficient (r) (*p* < 0.05)
P4-O2, Delta, Coherence	n.s *
F8-T4, Alpha, Coherence	n.s
F8-O1, Alpha, Coherence	0.164
C3-P4, Alpha, Coherence	n.s
T4-P3, Alpha, Coherence	0.293
Fp1-F7, Beta, Coherence	0.143
Fp2-F3, Beta, Coherence	n.s
Fp2-F7, Beta, Coherence	n.s
F3-F7, Beta, Coherence	n.s
F4-O2, Beta, Coherence	0.141
P4-O2, Beta, Coherence	n.s
O1-O2, Beta, Coherence	0.253
F3-F7, Gamma, Coherence	n.s
F4-P3, Gamma, Coherence	0.261
T5-O1, Gamma, Coherence	0.305
P3-O1, Gamma, Coherence	n.s
F3-T4, Theta, PLI	0.219
F4-T3, Theta, PLI	0.416
F7-T5, Beta, PLI	0.200
O1-O2, Beta, PLI	0.170

* non-significant difference.

**Table 8:** Results of the prediction of the MOCA score of DD patients using EO and EC features. Note: Bold values indicate the best performing results, while underlined values indicate the second-best results.

		SVR	ANNR	ENR
EO	*r* ^2^	0.54 ± 0.11	0.81 ± 0.09	**0.86 ± 0.04**
	MSE	1.04 ± 1.89	0.33 ± 0.83	**0.92 ± 1.75**
EC	*r* ^2^	0.75 ± 0.07	**0.88 ± 0.10**	0.82 ± 0.05
	MSE	1.16 ± 2.50	**0.33 ± 0.83**	0.92 ± 1.75

**Table 9:** Results of prediction of the MOCA score of DD patients using selected EC features by the NCA method. Note: Bold values indicate the best performing results, while underlined values indicate the second-best results.

		SVR	ANNR	ENR
NCA Selected Features from EC	*r* ^2^	0.43 ± 0.27	**0.89 ± 0.06**	0.76 ± 0.10
	MSE	1.31 ± 2.13	**0.23 ± 0.45**	0.73 ± 1.21

**Table 10:** Correlation Results for EC selected features by the NCA method in DD MOCA score prediction Task.

Features Name (Pathways, Sub-Band, Type)	Correlation Coefficient (r) (*p* < 0.05)
Fp2-F7, Delta, Coherence	0.254
O1-O2, Delta, Coherence	0.131
Fp1-O1, Alpha, Coherence	n.s *
T5-P3, Alpha, Coherence	−0.160
Fp2-F7, Beta, Coherence	0.114
F4-O2, Beta, Coherence	0.124
P4-O2, Beta, Coherence	0.110
F3-F7, Gamma, Coherence	0.173
T5-O1, Gamma, Coherence	n.s
O1-O2, Delta, PLI	n.s
F4-O2, Theta, PLI	n.s
F8-T4, Theta, PLI	n.s
T5-O1, Alpha, PLI	0.158
C3-T3, Beta, PLI	n.s
T3-T5, Beta, PLI	n.s
T3-O1, Beta, PLI	−0.211
T4-O1, Beta, PLI	−0.164

* non-significant difference.

## Data Availability

The data and analysis scripts supporting the findings of this study have been deposited in a public repository (Zenodo) under restricted access. The repository is available at https://doi.org/10.5281/zenodo.17955370 and is available as of 3 January 2026.
